# A remarkable clinical response in advanced gastric cancer treated with trastuzumab plus capecitabine combination chemotherapy: A report of two cases

**DOI:** 10.1002/ccr3.2056

**Published:** 2019-02-26

**Authors:** Naohiko Nakamura, Shinichi Kinami, Yoritaka Fujii, Seiko Miura, Jun Fujita, Daisuke Kaida, Yasuto Tomita, Takashi Miyata, Hideto Fujita, Nobuhiko Ueda, Takeo Kosaka

**Affiliations:** ^1^ Department of Surgical Oncology Kanazawa Medical University Hospital Uchinada, Kahoku Japan

**Keywords:** capecitabine, gastric cancer, trastuzumab

## Abstract

When trastuzumab + capecitabine and cisplatin chemotherapy could not be conducted continuously because of severe adverse reactions to cisplatin, trastuzumab + capecitabine could be an alternative systemic chemotherapy options for metastatic or recurrent gastric cancer patients.

## 1 BACKGROUND

Gastric cancer (GC) is the second leading cause of cancer‐related mortality and the fourth most commonly diagnosed malignant disease.^1^ Systemic chemotherapy is the standard treatment for recurrent or metastatic GC.^2^ Over the last decade, several new agents which show promising activity against GC have been identified.^3^ A randomized phase III trial examining the use of Trastuzumab (T‐mab) for Gastric Cancer (ToGA) was conducted as the first study demonstrating the efficacy of a target‐specific agent in advanced or metastatic GC that was positive for human epidermal growth factor receptor 2 (HER2) expression.^4^ Following the promising results of the ToGA trial, T‐mab combined with capecitabine and cisplatin (XP) is now a reference treatment for the first‐line treatment of HER2‐positive GC. However, the high dosage and long‐term administration of cisplatin can create severe systemic toxicity, including gastrointestinal problems, myelosuppression, and renal toxicity.^5^ As a result of systemic toxicity, continuation of a cisplatin combinatory regimen can sometimes be difficult for elderly patients or patients with several comorbidities.

We here presented two cases of metastatic or recurrent GC that showed a remarkable clinical response and resulted in an improved prognosis using long‐term T‐mab plus capecitabine combination chemotherapy without administration of cisplatin.

## 2 CASE PRESENTATIONS

### 2.1 Case 1

#### 2.1.1 Clinical data

**Figure 1:**
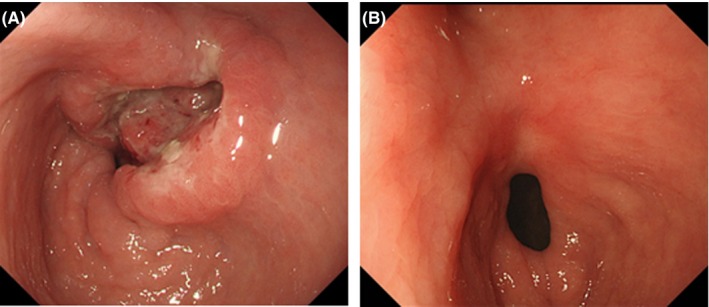
Upper gastrointestinal endoscopy findings. A, Type II advanced GC was identified in the lesser curvature of the antrum at the initial visit. B, Following trastuzumab plus capecitabine therapy, the primary tumor region showed remarkable regression

**Figure 2:**
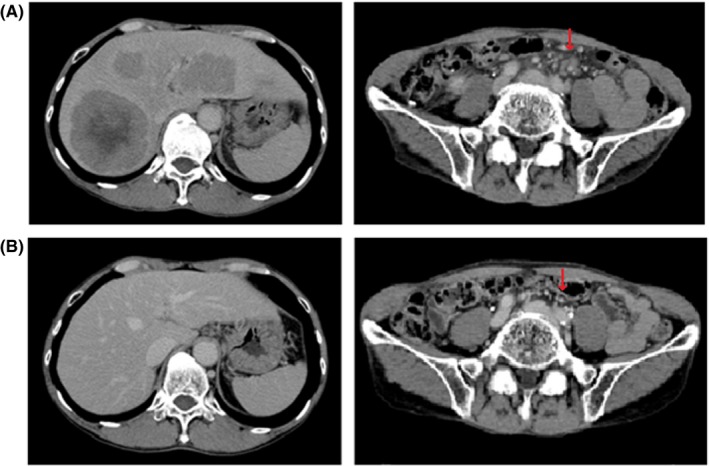
Enhanced computed tomography scan findings in the case 1. A, Multiple liver metastases in the both lobes and enlarged lymph nodes along the branch of superior mesenteric artery region (arrow) were identified before chemotherapy. B, Remarkable response of liver metastases and lymph node metastases were maintained using trastuzumab plus capecitabine therapy

**Figure 3:**
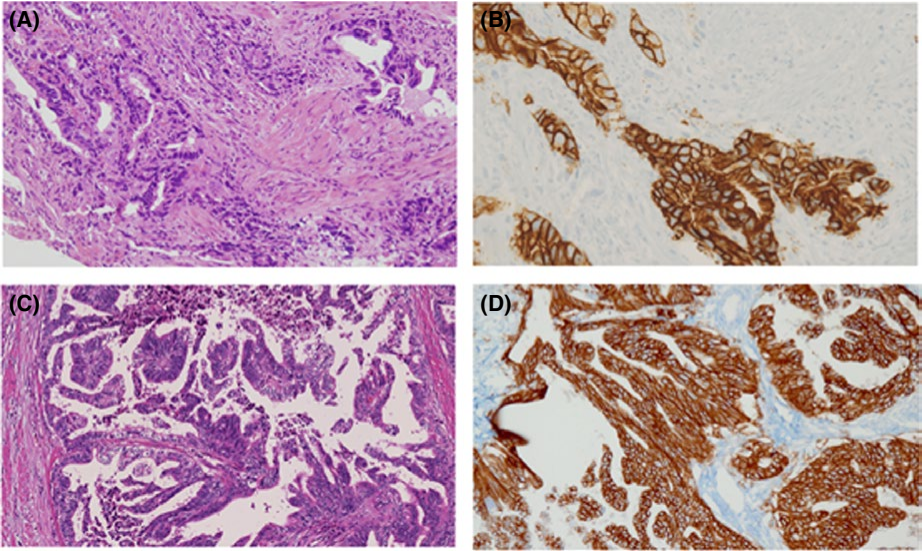
Histopathological findings in cases 1 and 2. Histological features of gastric tumor with hematoxylin and eosin staining (×10), and immunohistochemistry for human epidermal growth factor receptor 2 in tumor lesions (×20): case 1 (A, B) and case 2 (C, D), respectively

A 71‐year‐old male (height 157 cm, body weight 40.0 kg and performance status 1) who presented with anorexia and abnormal liver function following a blood examination was diagnosed with a type II advanced GC in the lesser curvature of the antrum after an upper gastrointestinal endoscopy (Figure 1). At the same time, an enhanced computed tomography scan (eCT) revealed multiple liver metastases and enlarged lymph nodes along the branch of superior mesenteric artery region (Figure 2A). Biopsy results from the gastric tumor yielded a diagnosis of a moderately differentiated adenocarcinoma (Figure 3A) and 3 + HER2 status by immunohistochemistry (IHC) (Figure 3B). The clinical diagnosis was L‐Less type 2 T3 N3 M1 H1 stage IVb (according to the 15th edition of Japanese classification of gastric cancer^6^).

#### 2.1.2 Treatment

For this patient, we chose to treat with T‐mab and XP chemotherapy; T‐mab was given by intravenous infusion at a dose of 8 mg/kg on day 1 of the cycle. Cisplatin 80 mg/m^2^ was given on day 1 by intravenous infusion. Capecitabine 1000 mg/m^2^ was given twice a day, orally, for 14 days followed by a 1‐week rest. Two weeks following the first treatment, impairment of renal function and anorexia were observed as adverse reaction to cisplatin; the estimated creatinine clearance was reduced to under 30 mL/min, and the severity of anorexia was categorized in grade 1 according to National Cancer Institute Common Terminology Criteria for Adverse Events version 3.0. Therefore, we terminated the administration of cisplatin and converted the regimen of systemic chemotherapy to T‐mab plus capecitabine combination therapy (T‐mab + capecitabine). After the start of T‐mab + capecitabine therapy, serum CEA was decreased rapidly and the multiple liver metastases, metastatic lymph nodes (Figure 2B), and primary tumor region showed a remarkable regression at the eighth course of T‐mab + capecitabine as a partial response due to RECIST ver. 1.1^7^ (Figure 1B).

#### 2.1.3 Outcome

The patient has survived for 40 months without progression of either liver metastases or primary tumor due to continuation of T‐mab + capecitabine chemotherapy.

### 2.2 Case 2

#### 2.2.1 Clinical Data

**Figure 4:**
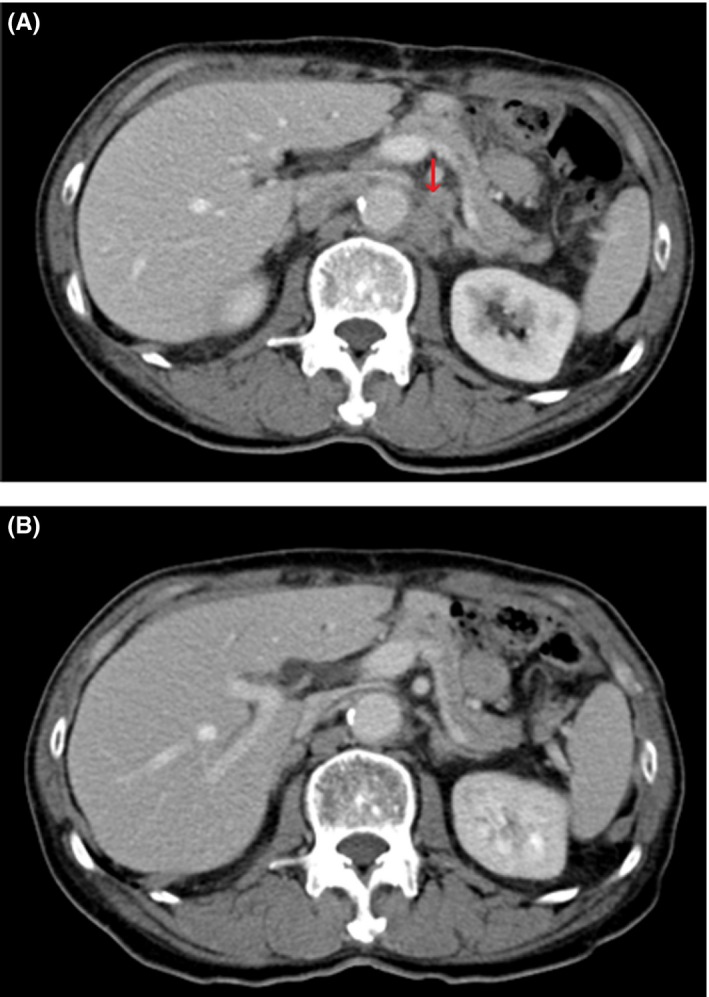
Enhanced computed tomography scan findings in case 2. A, Lymph node recurrences at the para‐aortic region were identified 4 months after surgery (arrow). B, Para‐aortic lymph node recurrences showed remarkable regression after trastuzumab plus capecitabine therapy

A 76‐year‐old male (height 164 cm, body weight 53.0 kg, and performance status 1) was referred to our hospital with a diagnosis of advanced GC. Upper gastrointestinal endoscopy indicated type III advanced GC in the lesser curvature of gastric body, and biopsy revealed a diagnosis of a papillary adenocarcinoma. Since lymph node metastasis that were closely located to the lesser curvature were suspected but distant metastasis of GC were not detected in preoperative imaging examinations, we performed an open total gastrectomy with lymph node dissection up to D2. The pathological stage was diagnosed as ML‐Less type 2 T4a N3 M0 stage IIIC^6^ and moderately differentiated adenocarcinoma with 3+ HER2 status in IHC results from the resected specimen (Figure 3C,D). Although adjuvant chemotherapy using oral intake of S‐1 was performed, lymph node recurrences at the para‐aortic region developed in an eCT at 4 months after surgery (Figure 4A).

#### 2.2.2 Treatment

To treat the recurrences, we started systemic chemotherapy with T‐mab plus paclitaxel as a protocol for a prospective clinical trial (JFMC 45‐1102); nevertheless, lymph nodes at the para‐aortic region had gradually enlarged. As a second‐line treatment, T‐mab with XP chemotherapy (same as the protocol in case 1) was conducted from 9 months following surgery. However, the patient showed grade 3 anorexia and fatigue in spite of the step‐by‐step dose reduction of both cisplatin and capecitabine. Thus, we applied the regiment of T‐mab + capecitabine combination therapy. Following five courses of T‐mab + capecitabine, the para‐aortic lymph node recurrences showed remarkable regression in the eCT (Figure 4B). It was estimated to be a complete response.^7^

#### 2.2.3 Outcome

**Figure 5:**
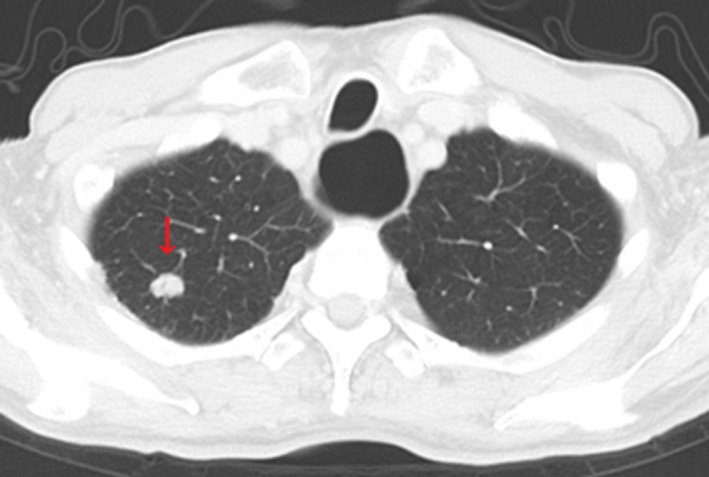
Chest computed tomography scan findings. A lung tumor was detected in the upper lobe of right lung 6 years after the surgery (arrow)

No adverse events and the progression of lymph node recurrences were not observed by T‐mab + capecitabine chemotherapy. However, a lung tumor was detected by eCT at 6 years after the surgery despite of continuation of T‐mab + capecitabine (Figure 5). For the lung tumor, a right lung lobectomy was performed. The postoperative histopathological examination revealed that the lung tumor was a metastasis of GC, and HER2 status was 3+ in IHC. The patient has survived for 6 years and 9 months after surgery without progression or any recurrences.

## 3 DISCUSSION

T‐mab has been integrated into the current standard chemotherapy for HER2‐overexpressing GC. The incidence of HER2 overexpression in gastric cancer is approaching 15%‐25%.^4^, ^8^ Although HER2 may be correlated to a poor prognosis, HER2 positivity is no longer considered a poor prognostic factor.^9^ In the ToGA study, T‐mab was found to improve the median OS when combined with XP or cisplatin/5‐FU (13.5 months) in comparison with XP or CF alone (11.1 months).^4^ Based on these results, cisplatin is recommended in combination with T‐mab as first‐line treatment for HER2‐positive GC. However, it is still unclear whether omitting administration of cisplatin in T‐mab‐based chemotherapy could a provide disadvantage for long‐term survival of HER2‐positive GC patients. We report here that continuation of T‐mab + capecitabine therapy achieved a remarkable clinical response and long‐term survival in metastatic or recurrent GC patients.

The ToGA study indicated that the overall tumor response rate was 47% and the complete response rate was 5%. In addition, the median progression‐free survival time was reported to be 6.7 months.^4^ In the present report, patients treated with T‐mab + capecitabine showed a remarkable clinical response for not only the primary cancerous region but also for liver or lymph node metastases. Of note, the long‐term uninterrupted administration of T‐mab + capecitabine without any adverse events could suppress the progression of metastasis and contribute to the long‐term survival of the patient. A previous report discussed a case of advanced GC with multiple liver metastases who reached more than 1 year of progression‐free survival using T‐mab + capecitabine chemotherapy.^10^ However, it has been not reported that continuation of T‐mab + capecitabine could sustain the disease control for a period of more than 3 years. Our cases may imply that an extremely small portion of HER2‐positive GC patients could show a better response for T‐mab‐based chemotherapy without cisplatin.

Cisplatin is one of the first‐line chemotherapy agents for treating advanced GC. Although many clinical trials have been conducted to uncover the best combinatory regimen of cisplatin with other chemotherapy agents such as docetaxel and fluorouracil, high dosage and long‐term administration of cisplatin sometimes causes severe adverse reactions.^11^ In the ToGA study, the proportion of patients who had anorexia, fatigue, and renal impairment as adverse events of T‐mab with XP chemotherapy was 46, 35, and 16%, respectively. As a result, the dose of cisplatin or capecitabine was reduced in nearly half of all patients following two courses of T‐mab with XP chemotherapy.^4^ In particular, dose reduction of cisplatin is recommended when creatinine clearance level is decreased under 40 mL/min. On the other hand, anorexia or fatigue caused by repeated utilization of chemotherapy agents could cause a deterioration in the patient's general condition and decrease the patient's quality of life. In our cases, renal impairment, anorexia, and fatigue were overcome by stopping the administration of cisplatin and patients were able to continue to undergo T‐mab + capecitabine chemotherapy for a long‐term period. Furthermore, in our second case, surgical resection could be performed safely for the lung metastasis after long‐term T‐mab + capecitabine chemotherapy. Therefore, it may be very important for multidisciplinary treatments for GC, including conversion surgery, to prevent a deterioration of patient's general condition caused by chemotherapy agent toxicity.

As potential limitations to current cases, it is unclear what the significance of continuation of T‐mab + capecitabine chemotherapy for control of disease progression following the response had been observed. A continuation of chemotherapy without adverse events, in some cases combining conversion surgery, seems to be necessary to aid long‐term survival at GC patients. However, it is difficult to make judgments on whether to change the chemotherapy regimen or attempt conversion surgery for regression regions when a remarkable clinical response is achieved. Further studies are needed to evaluate the benefit of T‐mab + capecitabine therapy as a multidisciplinary treatment option for recurrent or metastatic HER2‐positive GC.

In conclusion, we experienced two cases of metastatic or recurrent GC that showed a remarkable clinical response and obtained a better prognosis using long‐term T‐mab + capecitabine combination chemotherapy. When T‐mab + XP chemotherapy could not be conducted continuously because of severe adverse reactions to cisplatin, T‐mab + capecitabine could be an alternative systemic chemotherapy options for GC patients.

## CONFLICT OF INTEREST

The authors declare that they have no competing interests.

## AUTHOR CONTRIBUTION

SK and TK: involved in conceptualization. NN, YF, SM, JF, DK, YT, TM, HF, and NU: involved in data curation. NN and SK: involved in investigation. NN, SK, YF, SM, JF, DK, YT, TM, HF, and NU: performed the treatment. NN: drafted the manuscript. SK and TK: involved in revision. All authors have read and approved the final manuscript.
